# Post-Translational Modifications: Key “Regulators” of Pancreatic Cancer Malignant Phenotype—Advances in Mechanisms and Targeted Therapies

**DOI:** 10.3390/biomedicines13123013

**Published:** 2025-12-09

**Authors:** Yongkang Shi, Renyi Qin, Yiming Li

**Affiliations:** 1 Department of Biliary-Pancreatic Surgery, Affiliated Tongji Hospital, Tongji Medical College, Huazhong University of Science and Technology, 1095 Jiefang Ave., Wuhan 430030, China; shiyk_612@hust.edu.cn; 2 Department of Oncology, Affiliated Tongji Hospital, Tongji Medical College, Huazhong University of Science and Technology, 1095 Jiefang Ave., Wuhan 430030, China

**Keywords:** pancreatic cancer, post-translational modifications, phosphorylation, ubiquitination, targeted therapy, tumour microenvironment

## Abstract

Pancreatic cancer is a highly aggressive malignancy characterised by its invasive nature and poor therapeutic outcomes. These characteristics are closely associated with its complex biological characteristics and significant heterogeneity. Post-translational modifications (PTMs) have been identified as critical regulatory mechanisms through which cells respond to environmental changes and play a pivotal role in signal transduction. The various types of PTMs and their intricate regulatory mechanisms have a profound influence on multiple stages of pancreatic cancer progression. Research has demonstrated that PTMs modulate protein stability, activity, subcellular localization, and protein–protein interactions. The present review focuses on recent advances in our understanding of PTMs in pancreatic cancer, with a particular emphasis on phosphorylation, ubiquitination, SUMOylation, acetylation, lactylation, and O-GlcNAcylation. This study illuminates the molecular mechanisms and functional regulatory networks of PTMs within the distinctive tumour microenvironment of pancreatic cancer. Moreover, we summarise targeted therapeutic strategies directed at PTMs in pancreatic cancer to provide insights for future research and treatment development.

## 1. Introduction

Pancreatic cancer is a highly malignant tumour that has long been regarded as one of the deadliest cancers due to its highly invasive and insidious nature. Despite recent advances in diagnostic and treatment techniques, the overall survival rate in patients with these tumours remains significantly lower than that associated with other solid tumours [1]. Pancreatic cancer has surpassed breast cancer to become the third leading cause of cancer-related death [2], and its incidence is increasing in several countries [3]. Projections indicate that pancreatic cancer will be the second leading cause of cancer by 2040, surpassed only by lung cancer [4].

Surgical resection is the only radical treatment for pancreatic cancer, offering patients a reasonable chance of long-term survival [5]. However, it is important to note that only 15–20% of cases are resectable at initial diagnosis [6]. Despite the utilisation of adjuvant chemotherapy in conjunction with surgical resection—a strategy that has been demonstrated to enhance survival outcomes—the overall five-year survival rate for individuals diagnosed with pancreatic cancer remains at 13% [7]. The majority of patients present with advanced-stage disease or metastases at the time of diagnosis, thus undermining the opportunity for surgical intervention. Furthermore, patients who undergo radical resection still face a high recurrence risk, primarily due to residual micrometastases [8].

Regarding to the utilisation of pharmaceutical interventions, the molecular heterogeneity of pancreatic cancer [9] and the unique tumour microenvironment [10,11] constitute the biological basis of treatment resistance. Recent progress in targeted therapies and immunotherapy has been observed in other tumours, but these have yielded low response rates in pancreatic cancer [12]. Concurrently, the rise in the prevalence of obesity and the ageing population has been identified as a contributing factor to the escalating incidence of pancreatic cancer [13]. Consequently, there is an urgent need for the advancement of therapeutic interventions. Notably, the survival rate of patients diagnosed with pancreatic cancer has shown a gradual improvement [14], demonstrating the improvement of systemic therapeutic options for the disease, as well as the role of scientific research advances and multidisciplinary collaboration. However, this improvement is still confined to a small number of patients, and second-line treatment options for metastatic disease are extremely limited [15,16]. The crux of the issue lies in overcoming the clinical treatment dilemma of pancreatic cancer. This can be achieved through the identification of effective strategies for overcoming the tumour microenvironment barrier, the development of precise strategies based on molecular typing, the improvement of early diagnosis, and the optimisation of therapeutic options.

It is widely accepted that post-translational modifications (PTMs) are a pivotal regulatory mechanism for maintaining protein homeostasis and ensuring functional diversity in eukaryotes. To date, more than 450 unique protein modifications have been identified [17], including phosphorylation, acetylation, ubiquitination, and SUMOylation. These post-translational modifications have the capacity to alter the activity, intracellular distribution, and protein–protein interactions of target proteins [18,19]. The dynamic and reversible nature of these modifications—such as the dynamic and reversible regulation of kinases/phosphatases [20,21,22] and E3 ubiquitin ligases/deubiquitinating enzymes [23,24]—renders post-translational modifications pivotal to cellular responses to changes in the internal and external environment; furthermore, they play a central role in signal transduction. Recent studies have increasingly demonstrated that crosstalk exists between different PTMs, forming a multi-dimensional regulatory network [25]. A paradigmatic example is PD-L1, which is co-regulated by multiple PTMs [26]. This phenomenon underscores the necessity of summarizing the multi-dimensional regulatory patterns of PTMs in specific tumor types. Moreover, insights into the multi-dimensional regulatory effects of PTMs on their targets can provide valuable implications for the development of multi-target combination therapeutic strategies [27]. Against this background, PTMs are key drivers of pancreatic cancer progression due to their central role in regulating cellular signalling and protein function. This has led to a growing recognition of PTMs as promising targets for novel therapies.

Advances in technology and in-depth research have progressively unveiled the regulatory networks of various PTMs and their mechanistic roles in pancreatic cancer. This review systematically summarises the molecular mechanisms and functions of key PTMs, providing an in-depth analysis of their roles in the molecular pathogenesis of pancreatic cancer as well as recent progress in the development of PTM-targeted therapeutic strategies.

## 2. Phosphorylation: Regulatory Foundation of Oncogenic Signalling

Phosphorylation is the most prevalent and first-discovered post-translational modification [28,29], and can profoundly alter protein activities, particularly by regulating enzyme function [30]. This reversible modification is widespread in eukaryotes and constitutes a crucial post-translational regulatory mechanism. In mammalian cells, approximately one-third of all proteins are regulated by phosphorylation, a process maintained in dynamic balance through the coordinated actions of protein kinases and phosphatases [31]. Protein kinases covalently attach phosphate groups to serine, threonine, or tyrosine residues, whereas phosphatases remove them. Phosphorylation plays a key role in virtually all aspects of cellular life, participating in diverse processes such as transcription, differentiation, cell cycle progression, metabolism, and immune responses [32].

### 2.1. Mechanisms: Core Regulation by Kinases/Phosphatases

The specificity and dynamics of phosphorylation in pancreatic ductal adenocarcinoma (PDAC) are determined by the balance between kinase activation and phosphatase inhibition, with two pathways emerging as foundational: the KRAS-ERK cascade and STAT3 dual-site phosphorylation.

#### 2.1.1. KRAS-ERK: The “Master Switch” of Phosphorylation-Dependent Oncogenesis

Over 90% of PDACs harbour KRAS mutations (predominantly G12D, G12V, and G12R), which lock KRAS in a GTP-bound active state and trigger the downstream kinase cascade [33,34]. Mutational activation of RAS is a major driver of tumour progression in PDAC [35]. The RAF serine/threonine kinase family represents the most extensively studied direct effectors of KRAS [36]. Activated RAS-GTP binds to and promotes the activation of RAF kinase. Subsequently, activated RAF phosphorylates and activates the MEK1 and MEK2 protein kinases, which phosphorylate and activate the highly homologous ERK1 and ERK2 serine/threonine kinases [37,38]. This cascade consequently facilitates the development of pancreatic cancer.

A recent phosphoproteomic study further defined the ERK-dependent phosphoproteome in KRAS-mutant PDAC, identifying 4666 ERK-targeted phosphorylation sites across 2123 proteins [39]. This network underscores the pivotal function of ERK as a “central node” in the pathway, connecting KRAS mutations to the subsequent oncogenic outcomes observed in PDAC. Notably, ERK-mediated phosphorylation controls multiple key biological processes critical for PDAC progression. HIPK2 reduces ERK phosphorylation, thereby decreasing cMyc stability and inhibiting glycolysis in pancreatic cancer [40]. ERK-mediated phosphorylation also drives epithelial–mesenchymal transition (EMT): GINS2 specifically activates the ERK/MAPK signalling pathway to promote EMT [41]. Additionally, it contributes to therapeutic resistance through the ERK/Sp1 signalling cascade, which mediates the upregulation of Dicer expression in gemcitabine-resistant PDAC cells; targeting this pathway effectively reverses acquired gemcitabine resistance [42].

#### 2.1.2. STAT3 Dual-Site Phosphorylation: Integration of Inflammation and Proliferation

STAT3 is a critical transcription factor for the progression of PDAC and requires the coordinated phosphorylation of two residues. Tyr705 phosphorylation has been demonstrated to promote STAT3 dimerisation and nuclear translocation, thereby activating target genes involved in cell proliferation and apoptosis [43]. In addition, Ser727 phosphorylation enhances STAT3’s transcriptional efficiency and regulates its mitochondrial localisation [43]. Phosphorylation of STAT3 at Tyr705 drives its translocation into the nucleus, where it regulates a spectrum of tumour-associated biological processes, including angiogenesis, migration, invasion, cell proliferation, and the maintenance of cancer cell stemness [44]. In contrast, STAT3 phosphorylated at Ser727 localises to mitochondria, which modulate the activity of the electron transport chain, ultimately suppressing tumour cell apoptosis [44]. Recent research has revealed that upregulation of LCN2 by the RNA-binding protein BICC1 leads to the direct phosphorylation and activation of JAK2. This subsequently triggers the JAK2/STAT3 signalling pathway, promoting the secretion of the pro-angiogenic factor CXCL1. Targeting this pathway can inhibit tumour angiogenesis and enhance the efficacy of gemcitabine chemotherapy [45].

#### 2.1.3. Other Kinase–Phosphatase Axes

Beyond KRAS and STAT3, tissue-specific kinases further enrich the PDAC phosphorylation network by mediating context-dependent signalling cascades. VRK2 is a serine/threonine kinase that phosphorylates IKKβ at Ser177 and Ser181 to activate the TNFα-NF-κB pathway and drive the PDAC cell growth [46].

PKMYT1 is a cell cycle-regulating kinase in PDAC [47]. In addition to its conventional function of targeting CDK1 at Thr14/Tyr15 to inhibit CDK1 activity and impede the G2/M phase transition, researchers have found that PKMYT1 modulates PLK1 expression and phosphorylation (Tyr217), thus facilitating PDAC proliferation [47].

Meanwhile, AXL functions as a receptor tyrosine kinase that mediates TBK1 phosphorylation [48]. Following this, TBK1 activates AKT3 in an mTORC1-dependent manner, and this signalling cascade then drives the nuclear translocation of the EMT transcription factor Snail to ultimately enhance the invasive and metastatic potential of PDAC cells [48].

Furthermore, BLK drives the phosphorylation of FAM83A at Tyr138 [49]. This modification strengthens the interaction between β-catenin and TCF4; the complex then promotes β-catenin’s translocation into the nucleus and boosts its transcriptional activity, ultimately triggering the activation of the Wnt/β-catenin signalling pathway [49].

### 2.2. Pathological Regulation by Phosphorylation

#### 2.2.1. Proliferation and Metastasis by Phosphorylation

**Figure 1:**
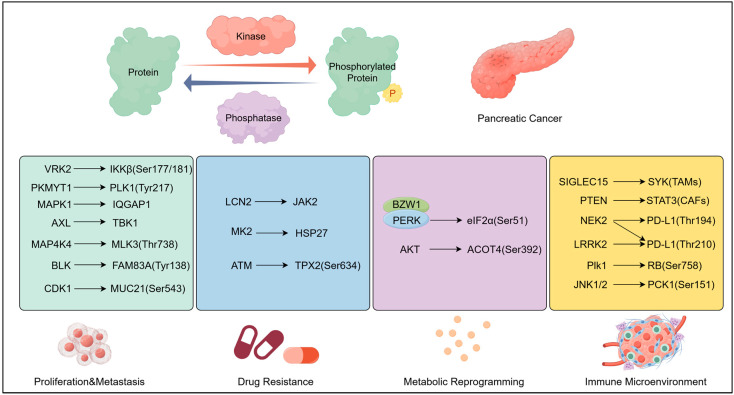
Schematic of protein phosphorylation-mediated regulatory networks in pancreatic cancer. This diagram illustrates how protein phosphorylation, catalysed by kinases and reversed by phosphatases, modulates multiple biological processes in pancreatic cancer.

Phosphorylation dysregulation permeates all key pathological processes of PDAC, with distinct kinase-phosphatase axes governing specific phenotypes (Figure 1). IQGAP1, a scaffolding protein that functions as a signalling hub to activate multiple pathways [50], was shown by Song et al. to be regulated through MAPK1 phosphorylation. Using combined phosphoproteomic and proteomic analyses, they demonstrated that this modification promotes the proliferation, migration, and invasion of pancreatic cancer cells [51]. MAP4K4 binds to MLK3 and promotes phosphorylation at its Thr738 site, thereby activating MLK3 kinase activity to drive pancreatic cancer progression [52].

Beyond intracellular signalling, phosphorylation also mediates intercellular crosstalk in PDAC. MUC21 is a key driver of perineural invasion (PNI) and metastasis in PDAC [53]. Schwann cell-derived GDNF phosphorylates MUC21 at the Ser543 site within PDAC cells via CDK1. Phosphorylated MUC21 then binds to RAC2, promoting activation of the JNK/ZEB1 pathway and inducing EMT. This mechanism reveals a novel model of nerve–tumour interactions in pancreatic cancer [53].

#### 2.2.2. Drug Resistance by Phosphorylation

Current standard chemotherapy regimens for pancreatic cancer include nab-paclitaxel plus gemcitabine [54], FOLFIRINOX [55], and NALIRIFOX [56]. The FOLFIRINOX chemotherapy regimen is currently an active and effective clinical strategy for pancreatic cancer. However, resistance to this regimen remains a challenge.

Recent research shows that BICC1-upregulated LCN2 mediates JAK2 phosphorylation and activation, triggering the JAK2/STAT3 pathway to promote CXCL1 secretion, thereby contributing to gemcitabine resistance [45].

Researchers have discovered that FOLFIRINOX treatment upregulates TNFα expression, which subsequently activates MK2, ultimately leading to significant phosphorylation and activation of HSP27 [57]. Targeting MK2 can block HSP27 phosphorylation and enhance FOLFIRINOX chemosensitivity. In animal models, the MK2 inhibitor ATI-450, combined with FOLFIRINOX, significantly improves efficacy without increasing toxicity [57]. Furthermore, Thao D Pham et al. found significantly elevated ERK phosphorylation levels in FOLFIRINOX-resistant KPC cell lines (generated by subjecting mouse PDAC cell lines to 6–8 cycles of FOLFIRINOX treatment) [58]. The MEK inhibitor trametinib significantly enhances the efficacy of anti-PD-1 therapy in syngeneic mouse models. These findings suggest the feasibility of combining trametinib with anti-PD-1 antibodies following FOLFIRINOX treatment.

PARP inhibitors are currently used as maintenance therapy for patients with metastatic pancreatic cancer harbouring germline BRCA1/2 mutations [59]. The choice of the DNA double-strand break (DSB) repair pathway is determined by the ATM-mediated phosphorylation of TPX2 at Ser634 [60]. Phosphorylated TPX2 activates Aurora A during mitosis to maintain genomic stability. Targeting the Ser634 site of TPX2 using cell-penetrating peptides can enhance the efficacy of PARP inhibitors in pancreatic cancer [60].

#### 2.2.3. Metabolic Reprogramming by Phosphorylation

In the metabolic reprogramming of pancreatic cancer, BZW1 acts as an adaptor protein for the PERK kinase, promoting the phosphorylation of eIF2α at Ser51 [61]. This activates the IRES-dependent translation pathway, upregulating HIF1α and c-Myc expression. This cascade enhances the Warburg effect and promotes pancreatic cancer progression. Researchers have also found that PERK/eIF2α phosphorylation inhibitors GSK2606414 and ISRIB significantly inhibit tumour growth and prolong survival [61]. Phosphorylation of Dicer (a cytoplasmic type III RNase) at S1728/S1852 promotes gemcitabine resistance in pancreatic cancer by activating glutamine metabolism and pyrimidine synthesis [62]. AKT-mediated phosphorylation of ACOT4 at Ser392 inhibits its binding to HSPA1A, leading to ACOT4 protein accumulation [63]. Consequently, excess ACOT4 generates CoA, promoting lipid metabolism in tumour cells.

#### 2.2.4. Immune Microenvironment Regulation

The role of phosphorylation modifications in regulating the pancreatic cancer immune microenvironment has garnered significant attention in recent years.

Tumour-associated macrophages (TAMs) and cancer-associated fibroblasts (CAFs)—two major components of the PDAC tumour microenvironment (TME)—are tightly regulated by phosphorylation to exert immunosuppressive effects. SIGLEC15, a novel TAM-related immune checkpoint, is expressed on both TAMs and PDAC cell surfaces [64]. The interaction between sialic acids expressed by PDAC cells and SIGLEC15 on TAMs induces SYK phosphorylation within the TAMs. This subsequently promotes the production of immunomodulatory cytokines, drives M2 macrophage polarisation, and accelerates the formation of an immunosuppressive pancreatic cancer microenvironment [64]. Therapeutic inhibition of SYK can significantly abolish this effect. In CAFs, loss of PTEN increases STAT3 phosphorylation. Activated STAT3 enhances CXCL1 secretion, which recruits myeloid-derived suppressor cells (MDSCs) to the TME—MDSCs then inhibit T cell proliferation and cytotoxicity, further compromising immune surveillance [65].

PD-L1, the most well-studied immune checkpoint in PDAC, is extensively regulated by phosphorylation at multiple residues, which dynamically modulates its stability and expression to evade PD-1/PD-L1 blockade. NEK2 phosphorylates PD-L1 at residues Thr194 and Thr210, blocking its ubiquitin–proteasome pathway degradation and thereby stabilising the protein [66]. This stabilisation undermines the efficacy of PD-L1-targeted immunotherapy and exacerbates the immunosuppressive microenvironment in pancreatic cancer, while targeting NEK2 can enhance anti-pancreatic cancer immune responses [66]. LRRK2, another kinase, also phosphorylates PD-L1 at Thr210 (a site overlapping with NEK2) to inhibit its degradation; in vivo studies have shown that the LRRK2 inhibitor adenosylcobalamin (AdoCbl) significantly enhances immunotherapeutic efficacy against PDAC [67]. Notably, these phosphorylation sites (Thr194/Thr210) are adjacent to Ser195—a residue whose phosphorylation induces aberrant PD-L1 glycosylation and promotes degradation [68]. Whether Thr194/Thr210 phosphorylation affects Ser195-mediated glycosylation via steric hindrance (thereby influencing PD-L1 stability) remains unclear, and further studies using techniques such as cryo-electron microscopy to resolve PD-L1’s structural changes are needed. Additionally, the functional crosstalk between NEK2 and LRRK2 also remains uncharacterised, including whether they act redundantly or sequentially: redundant action would mean the two kinases compensate for each other if one is inhibited, while sequential action would involve them coordinating to phosphorylate Thr210. Clarifying this relationship is essential for developing combination strategies, such as dual NEK2/LRRK2 inhibition, as such approaches could fully block PD-L1 stabilisation and further enhance immunotherapy efficacy.

Beyond PD-L1 stabilisation, phosphorylation regulates PD-L1 transcriptional expression via the NF-κB pathway: Plk1 kinase mediates RB protein phosphorylation at Ser758, which inhibits NF-κB nuclear translocation and suppresses NF-κB-dependent PD-L1 transcription [69]. Critically, this mechanism links Plk1 to immune checkpoint regulation—targeting Plk1 reduces PD-L1 expression and enhances immunotherapy efficacy in PDAC models, highlighting Plk1 as a potential combinatorial target for PDAC immunotherapy.

Phosphorylation also suppresses innate immune signalling in PDAC, further weakening anti-tumour immunity. The cGAS-STING pathway, a key mediator of innate immune activation, is inhibited by hypoxia-induced phosphorylation of PCK1 [70]. Hypoxia is a hallmark of the PDAC tumour microenvironment, and under such conditions, JNK1/2 kinases phosphorylate PCK1 at Ser151. This phosphorylation enhances the interaction between PCK1 and cGAS. Notably, PCK1 consumes GTP, a critical cofactor for cGAS activation, in a competitive manner. This interaction ultimately suppresses cGAS-STING signalling [70].

Collectively, phosphorylation modulates PDAC’s immune microenvironment through multi-layered mechanisms: it shapes the function of stromal/immune cells (TAMs, CAFs, and MDSCs), tunes immune checkpoint stability (PD-L1), and dampens innate immune signalling (cGAS-STING). Targeting these phosphorylation axes, either individually or in combination with immune checkpoint blockers (ICBs), has significant potential in converting the “cold” TME of PDAC into a “hot” one, thereby enhancing immunotherapy responses.

### 2.3. Targeted Therapy: Inhibitors of KRAS, STAT3, and Other Phosphorylation Targets

Phosphorylation modifications, as a core mechanism in cellular signalling regulation, play a key role in the initiation and progression of pancreatic cancer, as well as in the metabolic reprogramming of tumour cells and the remodelling of the tumour immune microenvironment. Consequently, their clinical translational potential and practical value have attracted significant attention. Thus, this section systematically summarises current advances in the development of targeted inhibitors for phosphorylation modifications and their role in pancreatic cancer therapy (Table 1).

#### 2.3.1. KRAS Inhibitors

KRAS mutations occur in over 90% of pancreatic cancers, with G12D (37.0%), G12V (28.2%), G12R (12.7%), and G12C (2.7%) being the most frequent subtypes [33,34]. KRAS was historically considered an “undruggable” target until the advent of the selective KRAS G12C inhibitor sotorasib (AMG-510), which offered new hope for lung cancer treatment [71]. Currently, sotorasib (AMG-510) and adagrasib (MRTX849) are FDA-approved for lung cancer. However, the relatively low mutation rate of KRAS G12C in pancreatic cancer (2.7%) [34] limits its broad applicability in PDAC. Consequently, more KRAS inhibitors are being discovered and intensively studied across multiple tumour types based on targeting various pathways.

The non-covalent KRAS G12D inhibitor MRTX1133 selectively blocks KRAS G12D activity with ultra-high affinity, inducing significant tumour regression in pancreatic cancer [72]. Crucially, researchers have further demonstrated that co-inhibiting compensatory pathways such as EGFR/PI3Kα enhances efficacy, providing the first validation of the feasibility of targeting KRAS G12D [73,74]. Although MRTX1133 shows therapeutic efficacy in preclinical studies, its effectiveness as a single agent against pancreatic cancer is suboptimal [74]. Compensatory activation of multiple pathways is a key reason for this limited efficacy, suggesting that combining MRTX1133 with specific targeted therapies may offer new therapeutic avenues.

Satoru Miyazaki et al. discovered that MRTX1133 feedback-activates STAT3 and ERK signalling pathways, resulting in resistance [75]. This demonstrates that JAK2/STAT3 and MAPK pathway reactivation are critical mechanisms of resistance to KRAS inhibitors [75]. Importantly, the MEK inhibitor trametinib and the JAK2 inhibitor fedratinib can effectively overcome resistance to both sotorasib and MRTX1133 [75]. Furthermore, MRTX1133—combined with the RAF/MEK dual inhibitor avutometinib [76], bedaquiline [77], the novel selective G9a degrader G9D-4 [78], or the ERBB inhibitor afatinib [79]—has also shown therapeutic potential, highlighting the significance of combining KRAS targeting with inhibition of multiple signalling pathways.

Additionally, researchers discovered that MRTX1133 demonstrates significant efficacy in both transplantable and orthotopic PDAC models with intact immune systems, leading to tumour regression [80,81,82]. Treatment induces substantial remodelling of the TME, including changes in fibroblasts, stroma, and macrophages. Critically, the authors emphasised the essential role of T cells in MRTX1133’s effects, confirming the T cell-dependent nature of this drug [80]. Using a KRAS G12D-driven PDAC model, Krishnan K Mahadevan et al. found that MRTX1133 inhibits early PDAC progression and increases intratumoural CD8^+^ T cell content [81]. In advanced PDAC, inhibiting KRAS G12D induced CD8^+^ T cell-mediated cancer cell death, and efficacy was further improved by combining with immune checkpoint inhibitors [81]. The KRASG12D inhibitor MRTX1133 shows model-dependent efficacy, with variable effects in 2D cell lines, enhanced efficacy in 3D cultures, tumour growth control in patient-derived xenografts (PDX), and regression in syngeneic models; mechanistically, it acts via ITGB1 and remodels the tumour microenvironment (enhancing IFN-γ signalling, recruiting effector CD8^+^ T cells), thus supporting KRAS G12D inhibition as a promising PDAC strategy [82]. These studies provide initial insights into immune microenvironment changes during MRTX1133 therapy, underscoring the importance of investigating KRAS inhibitor-induced TME alterations in future research.

Benjamin C Orsburn has published proteomic, metabolomic, and single-cell proteomic analyses of pancreatic cancer cell lines treated with MRTX1133 in public databases, serving as a valuable resource for subsequent metabolic studies [83].

Regarding the KRAS G12D inhibitor KS-58 [84], researchers successfully overcame its poor water solubility using nanocarrier technology [85]. The nanoparticle formulation demonstrated significant tumour targeting and sustained pharmacodynamic effects, effectively inhibiting the KRAS G12D downstream ERK signalling pathway [85]. This offers a novel nanomedicine solution with translational potential for treating refractory cancers harbouring RAS mutants.

Furthermore, another research team developed the pan-RAS inhibitor ADT-1004 [33], and validated its ability to inhibit growth and RAS activation in mouse PDAC models by reducing RAS activity and ERK phosphorylation levels. ADT-1004 also showed efficacy in PDX models carrying KRAS G12D, G12V, G12C, or G13Q mutations. Importantly, ADT-1004 demonstrated superior efficacy compared to the existing KRAS G12C inhibitors sotorasib and adagrasib, warranting anticipation for its broad-spectrum KRAS inhibitory effects [33].

Unlike the advanced development of KRAS G12C/G12D-specific inhibitors, no small-molecule inhibitors targeting KRAS G12V or G12R mutations have been approved, nor have related compounds entered registrational clinical trials. KRAS G12V/G12R mutants lack nucleophilically active residues like Cys in KRAS G12C, precluding the well-established covalent binding strategy for specific inhibitor-target anchoring. Instead, they require novel non-covalent designs to achieve targeted inhibition via precise recognition of the spatial conformation of mutated amino acids (Val and Arg) at position 12, significantly increasing drug development difficulty [86]. Meanwhile, KRAS mutants exhibit distinct hierarchical intrinsic GTP hydrolysis activity (G12C > G12D > G12V > G12R). With the lowest activity, KRAS G12V/G12R rarely switches spontaneously from a GTP-bound “active state” to GDP-bound “inactive state,” persisting in active conformations to drive oncogenic signalling [87], which impairs the binding efficiency of conformation-dependent inhibitors. Additionally, KRAS G12V and G12R mutations show lower sensitivity to the pan-KRAS inhibitor BI-2865 [88]. Their KRAS dependency, rate of transition to the GDP-bound state, and intrinsic GTP hydrolysis rate are significantly weaker than those of predominant mutation types such as G12C and G12D, resulting in the inability of drugs to exert effective effects through the inactive-state trapping mechanism [89].

#### 2.3.2. STAT3 Inhibitors

STAT3 is a critical therapeutic target in pancreatic cancer whose activation relies on the concerted phosphorylation of both Tyr705 and Ser727 residues [43]. Consequently, dual-targeting inhibitors are of significant importance for effectively inhibiting STAT3.

The natural compound Triptolide inhibits Tyr705 phosphorylation of STAT3, suppressing pancreatic cancer growth in tumour xenograft models [90]. WB436B, identified through virtual screening, significantly inhibits pancreatic cancer growth and metastasis in vivo [91]. Mechanistic studies reveal that WB436B binds to the SH2 domain of STAT3, and researchers have developed a novel dual-phosphorylation inhibitor lead compound, 4c, that also targets the STAT3 SH2 domain [92]. They found that 4c simultaneously blocks phosphorylation at both Tyr705 and Ser727 at nanomolar concentrations, effectively inhibiting both the nuclear transcriptional function and the mitochondrial oxidative phosphorylation (OXPHOS) function of STAT3. Compound 4c demonstrated promising therapeutic effects in both in vivo (subcutaneous model) and in vitro experiments [92]. Additionally, YY002, another dual-phosphorylation synchronous inhibitor, effectively suppresses pancreatic cancer growth and metastasis in preclinical models, exhibiting superior therapeutic efficacy compared with the clinical-stage STAT3 inhibitor BBI608 [43]. Both of these STAT3 inhibitors (4c and YY002) function by selectively binding to the STAT3 SH2 domain. This common mechanism provides a valuable framework for the further development of STAT3 inhibitors.

#### 2.3.3. Other Phosphorylation Targets

Researchers developed the highly potent oral IKKβ inhibitor quinoxaline urea analogue 84 through structural optimisation [93]. This compound targets IKKβ phosphorylation at Ser177/Ser181, blocking the TNFα-NF-κB signalling pathway. It maintains potent anti-tumour activity while significantly improving pharmacokinetic properties. In vivo studies confirmed that both monotherapy and combination with chemotherapy inhibit tumour progression [93]. JNK is aberrantly activated in pancreatic cancer, and Chen et al. demonstrated that abnormal JNK pathway activation upregulates inflammatory cytokines (IL-1β, IL-6, IL-8, and IL-15), showing that C66’s targeted inhibition of JNK phosphorylation blocks the formation of this inflammatory microenvironment, suppressing tumour proliferation and migration in vitro [94]. Innovative drug repositioning identified PF-3758309, initially developed as a PAK4 inhibitor, as having AMPK inhibitory activity [95]. This compound has been evaluated for its sensitivity and combinatorial therapeutic potential (with Erastin) in pre-clinical PDAC models, including cell models and patient-derived organoids (PDOs), and can overcome ferroptosis resistance in PDAC.

The MAP4K4-specific inhibitor GNE-495 exerts multiple therapeutic effects against pancreatic cancer, including inhibiting cell proliferation, inducing apoptosis, and reducing tumour stroma. Notably, it also decreases tumour burden and prolongs survival in KPC mice, a clinically relevant pancreatic cancer model [52]. Furthermore, F389-0746, a specific MAP4K4 inhibitor identified via virtual screening, significantly inhibits pancreatic cancer growth in subcutaneous models of nude mice and shows therapeutic potential [96]. CLK4 drives pancreatic cancer progression by phosphorylating the spliceosome. Researchers established a computational model integrating CLK4 inhibitor pharmacology and identified the novel specific CLK4 inhibitor compound 150441, which significantly inhibits pancreatic cancer cell growth and survival in cell models [97].

A multi-omics analysis strategy has revealed compensatory activation of the CDK kinase network in KRAS-mutant pancreatic cancer [98]. Screening an FDA drug library identified the multi-target CDK inhibitor AT7519, which simultaneously blocks phosphorylation in cell cycle regulation (CDK1/2) and transcriptional control (CDK7/9), inhibiting tumour growth in both PDX and PDO models [98]. Separately, Liu et al. found that SDCBP binds to YAP1 and inhibits its phosphorylation and subsequent β-TrCP-mediated degradation, thereby promoting pancreatic cancer progression [99]. Notably, zinc pyrithione (ZnPT) targets SDCBP, restoring YAP1 degradation and suppressing tumour growth in PDX and PDO models, indicating the SDCBP–YAP1 axis as a promising therapeutic target for pancreatic cancer. These findings validate PDX and PDO models as robust preclinical tools for identifying novel therapeutic agents and actionable pathways in pancreatic cancer, given their ability to recapitulate patient tumour heterogeneity and predict clinical responsiveness.

Mechanistically, phosphorylation modifications exhibit remarkable complexity and diversity in regulating target proteins, with primary functions including activating protease activity, modulating transcription factor activity, mediating intermolecular interactions, and regulating nuclear translocation processes. Conversely, for immune microenvironment regulation, phosphorylation predominantly targets PD-L1 through multiple pathways that actively modulate PD-L1, thereby influencing the immunosuppressive microenvironment in pancreatic cancer. Notably, targeting phosphorylation modifications can alter PD-L1’s ubiquitination or acetylation status, consequently affecting its protein stability to ultimately modulate pancreatic cancer’s immunologic landscape. Collectively, these mechanisms demonstrate that phosphorylation modifications play pivotal roles in pancreatic cancer pathogenesis, metabolic reprogramming, and immune microenvironment remodelling; therefore, therapeutic interventions targeting phosphorylation pathways represent a promising strategic direction for treatment.

**Table 1:** List of inhibitors targeting phosphorylation in PDAC.

Drug Name	Target Molecule	Pathway	Efficacy	PDAC Models	Ref.
MRTX1133	KRAS G12D	KRAS pathway	Inhibit pancreatic cancer growth	subcutaneous model	[72]
KS-58	KRAS G12D	KRAS/ERK pathway	Inhibit pancreatic cancer growth	subcutaneous/ orthotopic tumor model	[84,85]
ADT-1004	Pan-RAS (G12D/G12V/G12C)	Ras pathway; KRAS/ERK pathway	Inhibit pancreatic cancer growth	PDX	[33]
Triptolide	STAT3 (Tyr705)	STAT3 pathway	Inhibit pancreatic cancer growth	subcutaneous model	[90]
WB436B	STAT3 (SH2 domain)	STAT3 pathway	Inhibit pancreatic cancer growth and metastasis	subcutaneous model	[91]
Compound 4C	STAT3 (SH2 domain)	STAT3 pathway	Inhibits both STAT3 nuclear transcription and mitochondrial OXPHOS; Potent antitumor efficacy	subcutaneous model	[92]
YY002	STAT3 (SH2 domain)	STAT3 pathway	Inhibit pancreatic cancer growth and metastasis;	subcutaneous model	[43]
quinoxaline urea analog 84	IKKβ (Ser177/Ser181)	TNFα-NF-κB pathway	Both single-drug and combination chemotherapy can inhibit the progression of pancreatic cancer	subcutaneous model	[93]
C66	JNK	JNK pathway; Inflammatory cytokines	Disrupt the inflammatory microenvironment; Suppress tumour proliferation and migration	cell model	[94]
PF-3758309	AMPK	Ferroptosis	Sensitize pancreatic cancer cells to ferroptosis induction	PDO	[95]
GNE-495	MAP4K4	MAP4K4 pathway	Inhibit the proliferation and induce apoptosis; Reduce tumour stromal formation	KPC mouse model	[52]
F389-0746	MAP4K4	MAP4K4 pathway	Inhibit pancreatic cancer growth	subcutaneous model	[96]
Compound 150441	CLK4	CLK4-mediated spliceosome phosphorylation	Inhibits pancreatic cancer cell growth and survival	cell model	[97]
AT7519	CDK	Cell cycle; Transcriptional control	Block cell cycle and transcription process; Inhibit tumour growth	PDX and PDO	[98]
zinc pyrithione (ZnPT)	SDCBP	YAP1 phosphorylation	Inhibit pancreatic cancer growth	PDX and PDO	[99]

## 3. Ubiquitination: Regulator of Protein Homeostasis and Network Balance

### 3.1. Mechanisms: Specific Regulation of E3 Ligase/Deubiquitinase

The ubiquitin–proteasome system (UPS) specifically regulates protein stability and plays a pivotal role in the initiation and progression of pancreatic cancer. Recent studies indicate that ubiquitination modifies protein degradation processes and drives malignant progression in pancreatic cancer through multidimensional mechanisms. Ubiquitination specifically regulates protein homeostasis and function by adding ubiquitin or ubiquitin chains to substrate proteins, thereby playing a vital role in pancreatic cancer development. Protein ubiquitination is catalysed sequentially by three enzymes: E1 ubiquitin-activating enzymes, E2 ubiquitin-conjugating enzymes, and E3 ubiquitin ligases [100]. Within this pathway, E3 ubiquitin ligases specifically recognise substrates. Ubiquitination constitutes a dynamic and reversible process, with deubiquitinating enzymes (DUBs) removing ubiquitin or ubiquitin chains from substrate proteins to maintain proteomic homeostasis [101]. Approximately 100 deubiquitinating enzymes are currently known, categorised into five families: the ubiquitin-specific protease (USP) family, the ubiquitin C-terminal hydrolase (UCH) family, the ovarian tumour protease (OTU) family, the Josephin family, and the JAMM metalloprotease family [24]. Ubiquitin chains exhibit multiple configurations. The ubiquitin molecule itself contains seven lysine residues (K6, K11, K27, K29, K33, K48, and K63) and one N-terminal methionine (M1), which serve as sites for forming distinct ubiquitin chain types [102]. Each ubiquitin chain possesses unique structural and functional properties, thereby regulating protein stability, activity, or localisation [102].

**Figure 2:**
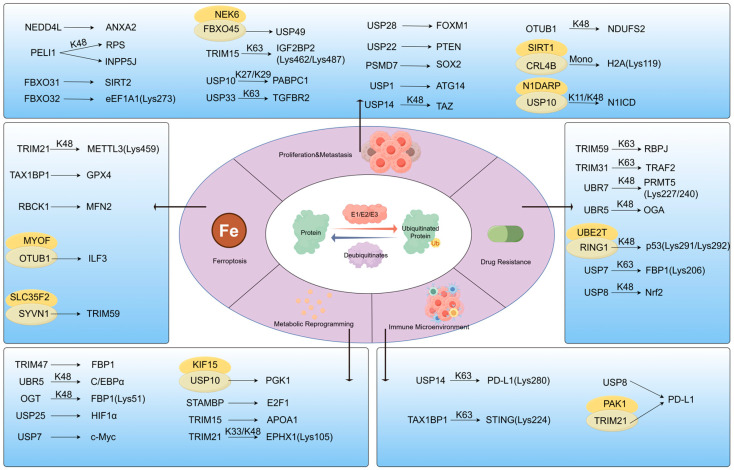
Schematic overview of ubiquitination—related regulatory networks in cancer. This diagram shows that ubiquitination, mediated by E1/E2/E3 enzymes and reversed by deubiquitinases, regulates multiple cancer-related biological processes, which are divided into proliferation and metastasis, ferroptosis, metabolic reprogramming, drug resistance, and the immune microenvironment.

Ubiquitination is extensively involved in the tumour immune microenvironment and proliferation, invasion, metastasis, and drug resistance, playing a crucial regulatory role in cancer progression. Accumulating experimental evidence has identified multiple E3 ligases and DUBs that exert key regulatory effects on pancreatic cancer progression. For instance, FBXL7 mediates ubiquitylation and proteasomal degradation of active c-SRC, suppressing EMT, cell invasion, and metastasis of prostate and pancreatic cancers [103]. On the DUB side, USP9X stabilises LATS kinase in the Hippo pathway by interacting with it, inhibiting YAP/TAZ nuclear translocation and suppressing pancreatic cancer progression [104]. This section focuses on recent advances in research concerning the regulatory processes of ubiquitination and deubiquitination (Figure 2).

### 3.2. Pathological Regulation by Ubiquitination

#### 3.2.1. Proliferation and Metastasis by Ubiquitination

E3 ubiquitin ligases have been identified as critical regulators in pancreatic cancer pathogenesis through their targeted ubiquitination and degradation of key substrates. Ubiquitin ligases regulate the stability or activity of substrate proteins by conjugating ubiquitin or ubiquitin chains to them. SDCBP inhibits the CK1δ/ε-mediated phosphorylation of YAP at Ser384/Ser387 by binding to YAP1, thereby blocking β-TrCP-dependent ubiquitin-mediated degradation of YAP1 and suppressing pancreatic cancer proliferation and metastasis [99]. NEDD4L promotes the ubiquitination of ANXA2, leading to its degradation and consequent suppression of tumour proliferation and metastasis [105]. PELI1 exerts pro-tumour effects through dual mechanisms: it facilitates K48-linked ubiquitination of RPS via its FHA domain (destabilising p53 [106]) and mediates degradation of the tumour suppressor INPP5J [107], collectively driving tumour progression. Importantly, this also implies that targeting PELI1 could simultaneously block both cascades, offering a more comprehensive and potent therapeutic strategy than inhibiting either pathway alone.

Multiple FBXO family members contribute to oncogenesis: FBXO31 mediates ubiquitination and degradation of SIRT2 [108]; FBXO32 catalyses the ubiquitination of eEF1A1 at Lys273, enhancing its activity [109]; and FBXO45—supported by NEK6 kinase—targets USP49 for degradation [110], synergistically enhancing tumour growth and invasiveness. Furthermore, TRIM15 induces K63-linked ubiquitination of IGF2BP2 at Lys462 and Lys487, augmenting its phase separation activity and subsequent stabilisation of TLR4 mRNA, thereby driving malignant progression [111].

DUBs play a pivotal role in pancreatic cancer progression by stabilising key oncogenic proteins and activating critical signalling pathways. USP10 enhances translation and tumour growth by removing K27/29-linked ubiquitin chains from PABPC1, facilitating its binding to CLK2 mRNA and eIF4G1 [112]. USP33 prevents lysosomal degradation of TGFBR2, leading to its accumulation and activation of TGF-β signalling to promote proliferation, migration, and invasion [113]. Several DUBs converge on stabilisation of transcriptional regulators: USP28 stabilises FOXM1 to enhance Wnt/β-catenin signalling and suppress apoptosis [114]; USP22 deubiquitinates and stabilises PTEN, upregulating p21 and promoting tumour growth [115]; and PSMD7 stabilises SOX2, thereby activating the Notch1 pathway [116]. Additionally, USP1 supports autophagy and growth through ATG14 stabilisation [117], while USP14 removes K48-linked ubiquitination from TAZ to promote invasion and metastasis, forming a positive feedback loop with TAZ [118]. OTUB1 also contributes to cell survival by removing K48-linked ubiquitination and stabilising NDUFS2, thereby suppressing mitochondrial cell death pathways [119].

Researchers discovered that the deacetylase SIRT1 interacts with the E3 ligase CRL4B to form a functional complex unit. This complex mediates H2ALys119 monoubiquitination, and its synergistic action promotes pancreatic cancer proliferation, autophagy, and invasive capacity [120]. Although ERK3 is incapable of directly phosphorylating Snail, it stabilises Snail by inhibiting its interaction with the specific E3 ubiquitin ligase FBXO11. In turn, this suppresses Snail ubiquitination and degradation, thereby enhancing the EMT [121]. Additionally, N1DARP competitively inhibits the binding between N1ICD and USP10, promoting K11 and K48 ubiquitin-mediated degradation of N1ICD [122], which consequently suppresses the Notch signalling pathway and reduces pancreatic cancer stem cell properties.

#### 3.2.2. Ferroptosis

The E3 ubiquitin ligase TRIM21 mediates proteasomal degradation of METTL3 via K48-linked ubiquitination at residue Lys459, thereby promoting ferroptosis in pancreatic cancer and enhancing antitumour immunity [123]. In multiple gastrointestinal tumours, Liu et al. reported that TRIM21 catalyses the K63-linked ubiquitination of FSP1 at residues Lys322 and Lys366, facilitating its membrane translocation and consequently suppressing ferroptosis [124]. Notably, the regulatory function of TRIM21 in ferroptosis extends beyond pancreatic cancer and exhibits context-dependent variability. For example, Sun et al. showed that in acute kidney injury models, TRIM21 promotes GPX4 ubiquitination and degradation, thereby inducing ferroptosis [125]. Conversely, Wei et al. found that MDM4 upregulates TRIM21, which inhibits the ubiquitination of GPX4 at Lys167 and Lys191 and shifts its ubiquitination profile from K48- to K63-linkage, thereby stabilising GPX4 and inhibiting ferroptosis [126]. Under HPAIV infection conditions, TRIM21 mediates the K63-linked polyubiquitination of SQSTM1, promoting ferroptosis through the SQSTM1–NRF2–KEAP1 axis [127]. These studies collectively demonstrate that TRIM21 plays multifaceted and sometimes opposing roles in ferroptosis regulation, suggesting that its pro- or anti-ferroptotic functions may depend on specific disease contexts and molecular subtypes.

Exogenous copper binds to the cysteine residues of GPX4, C107, and C148, inducing its ubiquitination and aggregation, thereby enhancing ferroptosis-mediated tumour suppression [128]. Then, the autophagy receptor TAX1BP1 facilitates GPX4 degradation, revealing a mechanistic link between copper induction and ferroptosis [128]. Under ferroptotic stress, RBCK1 mediates MFN2 ubiquitination and subsequent proteasomal degradation, thereby inhibiting ferroptosis in pancreatic cancer [129]. Separately, MYOF recruits OTUB1 and ILF3 to promote their interaction, which suppresses ubiquitin-mediated degradation of ILF3 [130]. The findings of this study demonstrate that stabilising ILF3 enhances the stability of LCN2 mRNA, thereby inhibiting ferroptosis in PDAC. SLC35F2 competitively binds to the E3 ubiquitin ligase SYVN1, which impedes TRIM59 degradation [131]. This stabilises TRIM59 and enhances its mediated degradation of p53, thereby inhibiting ferroptosis in pancreatic cancer. Together, these findings highlight the crucial roles of ubiquitination-related mechanisms in regulating ferroptosis in pancreatic cancer. Notably, how ferroptotic stress signals precisely modulate the activity, localization, or substrate specificity of specific ubiquitin ligases (e.g., RBCK1, SYVN1) and deubiquitinating enzymes (e.g., OTUB1) warrants in-depth investigation. Elucidation of this question will provide new insights into the crosstalk mechanisms between ferroptosis and the ubiquitination network.

#### 3.2.3. Drug Resistance by Ubiquitination

Ubiquitin ligases have been widely implicated in gemcitabine resistance in pancreatic cancer. TRIM59 stabilises RBPJ through K63-linked ubiquitination, while RBPJ transcriptionally upregulates TRIM59 expression, forming a positive feedback loop that reinforces gemcitabine resistance [132]. Catechin has been identified as a TRIM59 inhibitor that counteracts this resistance mechanism. Meanwhile, TRIM31 catalyses K63-linked ubiquitination of TRAF2, leading to p65 upregulation and subsequent activation of the NF-κB pathway, further promoting gemcitabine resistance [133]. Additionally, UBR7 mediates K48-linked ubiquitination (Lys227/Lys240) and degradation of PRMT5, contributing to resistance through modulation of glycolysis and the immune microenvironment [134]. Another E3 ligase, UBR5, enhances O-GlcNAcylation levels by targeting OGA—a key negative regulator of O-GlcNAcylation—for K48-linked ubiquitination and degradation, thereby inducing chemoresistance [135]. Beyond E3 ligases, the E2 conjugating enzyme UBE2T facilitates RING1-mediated K48-linked ubiquitination (Lys291/Lys292) and degradation of p53, which also promotes gemcitabine resistance [136]. On the deubiquitination side, USP7 removes FBP1 K63-linked ubiquitination (Lys206) and restricts its nuclear translocation, resulting in resistance to PARP inhibitors [137], whereas USP8 removes K48-linked ubiquitin chains from Nrf2, increasing its stability and fostering gemcitabine resistance [138].

#### 3.2.4. Metabolic Reprogramming by Ubiquitination

TRIM47 interacts with FBP1, leading to its ubiquitination and degradation, which promotes glycolysis and pancreatic tumour growth [139]. Furthermore, UBR5 mediates K48-linked ubiquitination of the transcription factor C/EBPα, thereby promoting its degradation and subsequent down-regulation of the glycolysis suppressor FBP1 [140]. This enhances aerobic glycolysis and accelerates pancreatic cancer progression [140]. Under hypoxic conditions, OGT induces O-GlcNAcylation at Ser47 of FBP1, which facilitates K48-linked ubiquitination at Lys51, thereby boosting glucose metabolism and illustrating the crosstalk between O-GlcNAcylation and ubiquitination [141].

In the highly hypoxic tumour microenvironment, USP25 deubiquitinates and stabilises HIF-1α, promoting glycolysis and supporting cancer cell survival under low oxygen, thereby driving pancreatic cancer progression [142]. USP7 stabilises c-Myc through deubiquitination, enhancing glycolytic flux and supporting tumour growth [143]. KIF15 recruits USP10 to deubiquitinate and stabilise PGK1, amplifying glycolysis in pancreatic cancer [144]. Additionally, STAMBP enhances the Warburg effect via PDK1 by deubiquitinating and stabilising E2F1, thereby promoting aerobic glycolysis and gemcitabine resistance [145].

While most studies emphasise glycolysis, lipid metabolism is emerging as a new target of ubiquitination in pancreatic cancer. TRIM15 mediates ubiquitination and degradation of APOA1, increasing lipid synthesis and promoting PDAC metastasis—suggesting that inhibiting triglyceride synthesis may represent a therapeutic strategy [146]. In arachidonic acid metabolism, TRIM21 catalyses K33/K48-linked ubiquitination and degradation of EPHX1 via Lys105, suppressing arachidonic acid metabolic pathways [147].

#### 3.2.5. Immune Microenvironment Remodelling

Although extensive studies have investigated PD-L1 ubiquitination in cancer, its regulatory mechanisms in pancreatic cancer remain to be systematically elucidated—with critical gaps in understanding the interplay between ubiquitination, phosphorylation, and autophagic degradation in shaping PD-L1 expression and immunotherapy responses. Specifically, USP14 downregulates PD-L1 in pancreatic cancer by removing K63-linked ubiquitination at Lys280, thereby enhancing antitumour immunity [148], while USP8 stabilises PD-L1 through deubiquitination to promote immune suppression [149]—highlighting the functional dichotomy of DUBs in PD-L1 regulation, which may depend on tumour context or substrate modification patterns. NEK2 phosphorylates PD-L1 at Thr194/Thr210 to shield it from ubiquitin-mediated degradation [66]. PAK1 competitively binds to PD-L1, preventing its interaction with the E3 ligase TRIM21 and subsequent ubiquitination/degradation, and combined inhibition of PAK1 with anti-PD-1 therapy significantly improves pancreatic cancer treatment outcomes [150]—underscoring the potential of targeting PD-L1 stability regulators as combinatorial strategies to overcome immunotherapy resistance. Additionally, the autophagy receptor TAX1BP1 mediates K63-linked ubiquitination of STING at Lys224, triggering its autophagic degradation to dampen immunotherapy efficacy [151]; targeting this TAX1BP1-STING axis thus emerges as a promising approach to restore anti-tumour immunity. Collectively, these findings reveal a complex network of post-translational modifications (ubiquitination, phosphorylation) and degradation pathways (proteasomal, autophagic) that fine-tune PD-L1/STING levels in pancreatic cancer, but future studies should focus on identifying the elusive E3 ligases for PD-L1, clarifying how DUBs achieve substrate specificity, and exploring whether targeting multiple nodes of this regulatory network (e.g., PAK1 + TAX1BP1) can synergistically enhance immunotherapy responses in pancreatic cancer—an area that may address the inherent resistance of this malignancy to immune checkpoint blockade.

### 3.3. Ubiquitination-Targeted Inhibitors: Applications and Research

Recent studies have identified multiple inhibitors targeting ubiquitin modification, which we summarise here to facilitate subsequent research (Table 2). Inducing ferroptosis has emerged as a promising therapeutic strategy against tumours; however, conventional approaches often cause collateral damage to immune cells during cancer cell ferroptosis, thereby compromising antitumour immunity. To address this, N6F11 has been identified as a compound that specifically triggers ferroptosis in pancreatic cancer, as validated in both KPC mice and the orthotopic tumour model [152]. Mechanistically, N6F11 binds to the RING domain of TRIM25, promoting TRIM25-mediated K48-linked ubiquitination and degradation of GPX4. Importantly, N6F11 also activates CD8^+^ T cell-mediated anti-tumour immunity, offering a new strategy for ferroptosis-based immunotherapy [152].

Regarding E2/E3-targeted inhibitors, Jiang et al. performed organoid-based screening and identified the UBE2T inhibitor PGG, which significantly sensitises pancreatic cancer to gemcitabine. This effect was validated in KPC mouse and PDX models, where PGG markedly suppressed tumour growth and extended survival [136]. Taraxasterol (TAX) promotes MDM2 ubiquitination and degradation, facilitating p53 nuclear translocation and inhibiting pancreatic cancer cell proliferation [153]. Through virtual screening, DHPO has been identified as an inhibitor of the E2-conjugating enzyme UbcH5c [154]. It blocks UbcH5c-mediated degradation of IκBα, and its antitumour efficacy has been confirmed in orthotopic models using SW1990 and PANC-1 cells. Notably, Guan et al. also identified DHPO via virtual screening but as a USP7 inhibitor that induces ferroptosis in gastric cancer [155]. This dual activity raises critical questions about DHPO’s target specificity, particularly whether it can simultaneously inhibit tumour growth and induce ferroptosis in the same cancer type—highlighting the need for further validation of its molecular targets to support clinical translation.

In the context of DUB inhibitors, Yang et al. revealed that USP8 stabilises PD-L1 through deubiquitination, and combining USP8 inhibitor DUB-IN-2 with anti-PD-L1 therapy potently suppresses pancreatic tumour growth, as demonstrated in the subcutaneous model [149]. The USP1 inhibitor I-138 significantly suppresses pancreatic cancer growth and enhances the efficacy of cisplatin in PDAC, supporting its use as an autophagy-activating strategy to overcome chemotherapy resistance [117]. Multiple USP1 inhibitors are currently under development, including ML323, RO7623066, TNG348, XL309–101, SIM0501, and HSK39775 [156], warranting further comparison of their functional specificity and efficacy.

Similarly, USP7 promotes PDAC growth through glycolysis by stabilising c-Myc. Treatment with the USP7 inhibitor P5091 significantly delayed tumour progression in KPC mice [143]. Although over twenty USP7 inhibitors have been reported to date, most have been functionally validated only in limited tumour models [157]. Thus, targeting USP7 remains a highly promising therapeutic strategy for pancreatic cancer.

**Table 2:** List of inhibitors targeting ubiquitination in PDAC.

Drug Name	Target Molecule	Pathway	Efficacy	PDAC Models	Ref.
N6F11	TRIM25	Ferroptosis; Anti-tumour immunity	Trigger ferroptosis in tumour; Activate antitumour immunity	KPC and orthotopic tumor model	[152]
PGG	UBE2T	Ubiquitination-dependent degradation of p53	Enhance the therapeutic sensitivity to gemcitabine; Inhibition of tumour growth	PDX and KPC	[136]
Taraxasterol (TAX)	MDM2	Promoting nuclear translocation of p53	Inhibit pancreatic cancer growth	subcutaneous model	[153]
DHPO	UbcH5c	IκBα degradation and NF-κB activation	Inhibit pancreatic cancer growth and metastasis	orthotopic tumor model	[154]
DUB-IN-2	USP8	Anti-tumour immunity	Enhance the antitumor effect of PD-L1 antibodies	subcutaneous model	[149]
I-138	USP1	Autophagy	Inhibit PDAC progression and enhances cisplatin efficacy	subcutaneous model	[117]
P5091	USP7	glycolysis	Inhibit pancreatic cancer progression	KPC and subcutaneous model	[143]

## 4. Emerging PTMs as Network Extensions

**Figure 3:**
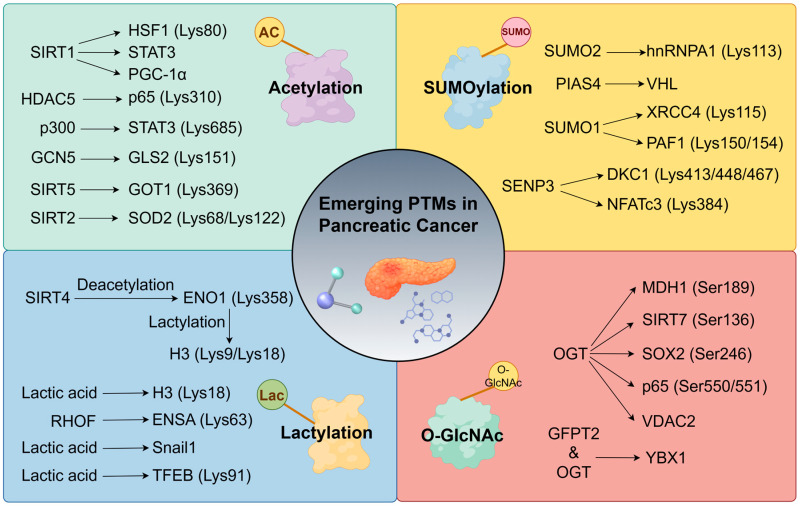
Schematic of emerging protein post-translational modifications (PTMs) in pancreatic cancer. This diagram showcases that emerging PTMs regulating pancreatic cancer are divided into four categories: acetylation, SUMOylation, lactylation, and O-GlcNAc.

The preceding section systematically outlined the regulatory mechanisms of phosphorylation and ubiquitination modifications in pathogenesis, drug resistance, tumour microenvironment metabolism, and immune remodelling in the context of pancreatic cancer, alongside the application of inhibitors. However, the malignant regulatory networks in pancreatic cancer are highly complex. Beyond these two core modifications, other emerging post-translational modifications of proteins also play a significant role in pancreatic cancer. This section further refines the research framework for post-translational modification regulation in pancreatic cancer and summarises the progress and applications of their inhibitors (Figure 3 and Table 3).

### 4.1. Acetylation: A Regulator of Immune Microenvironment/Metabolism

#### 4.1.1. Immune Microenvironment Regulation

Protein acetylation is a key post-translational modification that participates extensively in diverse disease processes by regulating protein stabilisation, function, localisation, and enzymatic activity [158].

In pancreatic cancer immunoregulation, both acetylation and deacetylation modifications play pivotal roles. Specifically, BAP1 deficiency induces immune tolerance in pancreatic cancer [159]. The mechanism involves BAP1 competitively binding the Lys80 site of HSF1 with the deacetylase SIRT1, thereby maintaining HSF1 in an acetylated state. This acetylation promotes HSF1 interaction with HSP70, leading to HSF1 dissociation from chromatin and ultimately enhancing immunotherapy efficacy. Notably, approximately 27% of PDAC patients exhibit BAP1 loss, and targeted inhibition of SIRT1 has been demonstrated to enhance immunotherapy response in such BAP1-deficient PDAC [159].

Regarding immune checkpoint regulation, HDAC5 inhibits the interaction between the transcription factor p65 and coactivators such as BRD4 by deacetylating the Lys310 site of p65, thereby reducing the expression levels of PD-L1 [160]. Targeted inhibition of HDAC5 significantly enhances pancreatic cancer response to immune checkpoint blockade therapy. Notably, a critical regulatory point is the phosphorylation of p65 at Ser311; this modification inhibits HDAC5-p65 interaction, thereby promoting p65 acetylation [160].

The activity of the STAT3 signalling pathway is also significantly regulated by acetylation modifications. The circular RNA circPTPN22 enhances STAT3 acetylation by inhibiting its interaction with the deacetylase SIRT1, which promotes cancer progression and suppresses antitumour immunity within the pancreatic cancer microenvironment [161]. Furthermore, the long non-coding RNA FGD5-AS1, originating from exosomes, interacts with the histone acetyltransferase p300 to mediate acetylation at the Lys685 site of STAT3. This modification activates the STAT3/NF-κB signalling pathway and induces macrophage M2-type polarisation, thereby promoting pancreatic cancer progression [162].

Hypoxia within the tumour microenvironment is also a crucial regulatory factor. Hypoxia induces acetylation at the Lys151 site of GLS2 via the acetyltransferase GCN5, which enhances GLS2’s interaction with YAP1 [163]. Concurrently, TTLL1 mediates glutamylation at YAP1’s E100 site—the GLS2–YAP1 interaction and YAP1 glutamylation act synergistically to promote the nuclear translocation of YAP1 and its transcriptional activation function, ultimately leading to PD-L1 upregulation and enabling pancreatic cancer cells to achieve immune evasion.

#### 4.1.2. Metabolic Reprogramming

Under high glucose stimulation, the transcription factor YY1 induces the upregulation of long intergenic non-coding RNA LINC00842 [164]. LINC00842 competitively binds to the PGC-1α, thereby obstructing the deacetylation of PGC-1α mediated by the deacetylase SIRT1 and maintaining the acetylated state of PGC-1α. This modification drives pancreatic cancer cell metabolic reprogramming toward lipid metabolism, enhancing their malignant phenotype [164].

CAFs in the tumour microenvironment regulate pancreatic cancer cell metabolism by secreting acetate. Research indicates that the ACSS2 pathway mediates acetylation modification at the Lys19 site of transcription factor SP1 [165]. This modification enhances SP1’s protein stability and transcriptional activity, conferring the ability to survive in acidic environments to pancreatic cancer cells.

SIRT5 catalyses deacetylation at position Lys369 of GOT1, reducing GOT1 enzyme activity [166]. This subsequently inhibits glutamine metabolism and glutathione synthesis, ultimately suppressing pancreatic cancer growth. Based on this mechanism, researchers developed the SIRT5 selective activator MC3138. This activator significantly reduces the acetylation level of the GOT1 protein and inhibits its enzymatic activity. Notably, the combination of MC3138 with gemcitabine significantly improves the precision treatment strategy for SIRT5-low PDAC.

#### 4.1.3. Acetylation Target Therapy

Targeting protein acetylation modifications offers novel therapeutic avenues for pancreatic cancer, with multiple compounds demonstrating therapeutic potential by influencing specific acetylation modifications. Firstly, the natural flavonoid fisetin exhibits a dual mechanism of action: on one hand, it inhibits pancreatic cancer stem cell properties and enhances gemcitabine sensitivity, which has been validated in both the KPC model and the subcutaneous model, by increasing acetylation levels at the Lys33 site of CDK1, thereby suppressing its phosphorylation [167]; concurrently, it promotes acetylation at sites Lys68 and Lys122 of SOD2 by suppressing the expression of the deacetylase SIRT2, thereby inhibiting pancreatic cancer cell proliferation and inducing apoptosis [168]. Regarding histone acetyltransferase (HAT) targeting, the inhibitor C646 effectively suppresses acetylation of histone H3 at lysine 9 (H3K9) and lysine 27 (H3K27), demonstrating favourable therapeutic effects in pancreatic cancer subcutaneous models [169]. For deacetylase SIRT6 targeting, researchers identified the novel pyrrole–pyridine–imidazole derivative 8a as an effective inhibitor [170]. This compound significantly elevates intracellular histone H3 acetylation levels, inhibits pancreatic cancer cell proliferation, promotes apoptosis, and effectively reverses resistance to gemcitabine. Histone deacetylase inhibitors, including Givinostat and Dacinostat, significantly enhance pancreatic cancer cell sensitivity to CTL-mediated killing [171]. Furthermore, the inhibitor ISOX, by targeting the deacetylase HDAC6, promotes non-histone acetylation modifications of the proto-oncogene c-Myc, leading to reduced c-Myc protein stability and thereby inhibiting pancreatic cancer stem cell properties, tumour growth, and metastasis [172]. Notably, the combination therapy strategy of ISOX with 5-fluorouracil (5FU) has demonstrated significant synergistic antitumour effects in both tumour organoids and mouse models harbouring the Kras G12D mutation [172].

### 4.2. SUMOylation

SUMOylation is a post-translational modification where small ubiquitin-like modifiers (SUMOs) bind covalently and reversibly to target proteins. In mammals, four isoforms (SUMO-1/-2/-3/-4) exist, which are critical for regulating nuclear organisation and cell viability. Their expression is markedly upregulated in carcinogenesis-related processes, including cell growth, differentiation, senescence, oxidative stress, and apoptosis [173].

The KRAS-G12D mutation induces hyperactivation of the SUMOylation pathway in pancreatic cancer cells. This activation drives SUMO2 binding to the Lys113 site of hnRNPA1, promoting interaction between hnRNPA1 and the transport-associated protein TSG101 [174]. This subsequently enhances the packaging of hnRNPA1 into extracellular vesicles (EVs) and its secretion. These SUMO-modified EV-hnRNPA1 particles stabilise transcripts of the heterobox protein PROX1, ultimately driving lymphangiogenesis and lymph node metastasis in pancreatic cancer [174]. Concurrently, the SUMO ligase PIAS4 mediates SUMOylation of the VHL, inducing VHL oligomerisation and loss of its normal function, which promotes pancreatic cancer growth [175].

SUMOylation profoundly influences pancreatic cancer’s response to radiotherapy and chemotherapy. The circular RNA circBIRC6 directly binds XRCC4 to promote its SUMOylation, facilitating the interaction between the Lys115 site of XRCC4 and SUMO1 [176]. This modification enhances XRCC4’s chromatin localisation, mediating oxaliplatin resistance in pancreatic cancer cells. Notably, the extent of PML protein’s own SUMOylation strongly correlates with both gemcitabine and oxaliplatin resistance in pancreatic cancer [177]. Under radiotherapy, SUMO1 modifies PAF1 at residues Lys150 and Lys154, enhancing PAF1’s interaction with PML [178]. This promotes the survival and growth of PDAC cells post-radiotherapy.

Among de-SUMOylating enzymes, SENP3 has been most extensively studied, reflecting its critical role in pancreatic cancer—and its function is dual-faceted. SENP3 binds to DKC1, mediating its de-SUMOylation and accelerating its proteolytic degradation [179]. This disrupts DKC1’s interaction with snoRNP proteins, thereby inhibiting invasion and metastasis in the context of pancreatic cancer. Conversely, under hypoxic stress, dephosphorylation of NFATc3 promotes its nuclear translocation and activates target gene expression; during this process, SENP3 mediates de-SUMOylation at NFATc3’s Lys384 site and disrupts its binding to protein kinase GSK-3β [180]. This reduces NFATc3 phosphorylation, further promoting its nuclear translocation and functional activation to support pancreatic cancer cell survival [180]. Clearly, SENP3 promotes pancreatic cancer cell survival under hypoxia while inhibiting metastasis; its dual oncogenic and tumour-suppressive roles remain unclear and warrant further investigation.

For SUMOylation-targeted therapy, Sumit Kumar and colleagues validated the novel SUMO ligase E1 inhibitor TAK-981 in a PDAC mouse model [181]. Notably, TAK-981 also exhibited potent tumour-suppressive effects in acute myeloid leukaemia [182,183]. The findings show that low concentrations of TAK-981 effectively inhibit SUMOylation in PDAC, with a dual antitumour mechanism: it impedes tumour progression by blocking cell cycle progression while simultaneously activating the interferon signalling pathway to enhance antitumour immune responses [181]. Additionally, regarding the link between the SUMOylation pathway and the MYC oncogene, researchers found that the small-molecule inhibitors of SUMO ligase (SAE)—namely, ML-792 and ML-93—can mediate G2/M phase cell cycle arrest and induce apoptosis, thereby effectively inhibiting pancreatic cancer growth as validated in both the tumour organoid model and the subcutaneous model [184].

### 4.3. Lactylation

Lactylation (Kla) is a novel post-translational modification that was first identified in 2019 on histone lysine residues, characterised by a mass shift of 72.021 Da. It can be promoted by endogenous or exogenous L-lactate and is regulated by cellular metabolic states such as glycolysis. Kla occurs via enzymatic or non-enzymatic mechanisms and involves “writer”, “eraser”, and “reader” proteins, playing important roles in signalling transduction and biological regulation [185].

SIRT4 mediates deacetylation at the Lys358 site of ENO1. This deacetylation induces lactylation at histone H3 of the K9 and K18 sites, thereby sustaining pancreatic cancer stemness [186]. Another study by Li et al. isolated perineuronal infiltration-associated cancer-associated fibroblasts (pCAFs) [187]. Lactic acid secreted by these pCAFs, upon uptake by pancreatic cancer cells, promotes lactylation at histone H3K18, thereby driving perineuronal infiltration in pancreatic cancer [187]. In TAMs, VSIG4 deficiency markedly reduces histone H3K18 lactylation levels, diminishing SPP1 transcription and disrupting the interaction network between TAMs and neutrophils [188]. Beyond TAMs, elevated global protein lactylation in PDAC also correlates with poor prognosis. Specifically, high lactate concentrations induce K63-linked lactylation of ENSA, activating the STAT3/CCL2 signalling pathway to recruit tumour-associated macrophages and ultimately induce immunotherapy resistance [189]. RHOF promotes Snail1 lactylation and nuclear translocation, thereby enhancing pancreatic cancer growth, migration, and invasion [190]. Furthermore, lactate molecules covalently modify TFEB. Lactylation at the Lys91 site of TFEB inhibits its interaction with WWP2, stabilising TFEB by obstructing WWP2-mediated proteasomal degradation pathways and thereby promoting autophagy in pancreatic cancer [191].

### 4.4. O-GlcNAcylation

O-GlcNAcylation is a reversible post-translational modification that was discovered over 40 years ago [192], primarily occurring on serine/threonine residues of proteins. It is catalysed by O-GlcNAcyltransferases (OGTs) [193,194] and removed by O-GlcNAc hydrolases (OGAs) [195].

O-GlcNAc modification at the Ser189 site of the MDH1 enhances its enzymatic activity, thereby promoting glutamine metabolism and supporting tumour growth [196].

OGT promotes pancreatic cancer progression by stabilising SIRT7 and enhancing its deacetylation of histone H3K18, which suppresses tumour suppressor gene transcription [197]. This process is regulated by O-GlcNAcylation at SIRT7’s Ser136 site. Simultaneously, OGT-mediated modification of SOX2 at Ser246 stabilises its nuclear localisation, further supporting tumour development [198]. The small-molecule inhibitor OSMI-1 effectively targets OGT and suppresses these oncogenic mechanisms [198].

Recent research has established a workflow for analysing O-GlcNAc modifications, identifying 2831 such sites in PANC-1 cells [199]. This analysis also innovatively discovered that Tyr residues undergo O-GlcNAcylation [199]. Within key pancreatic cancer signalling pathways, O-GlcNAc modification at Ser550/Ser551 of the NF-κB p65 subunit promotes nuclear translocation, phosphorylation, and transcriptional activity, thereby accelerating tumour proliferation and migration [200]. Moreover, interferon-induced protein IFIT3 binds to the mitochondrial channel protein VDAC2 and promotes its interaction with OGT, inducing O-GlcNAcylation of VDAC2 and ultimately conferring gemcitabine resistance in PDAC [201].

The metabolic enzyme GFPT2 facilitates the O-GlcNAc modification and nuclear translocation of the transcription factor YBX1, activating IL18 transcription [202]. This promotes M2-type polarisation of tumour-associated macrophages, contributing to an immunosuppressive tumour microenvironment.

**Table 3:** List of inhibitors targeting other PTMs in PDAC.

Drug Name	Target Molecule	Pathway	Efficacy	PDAC Models	Ref.
Fisetin	CDK1	Stem cell properties	Enhance gemcitabine sensitivity	KPC; subcutaneous model	[167]
Fisetin	SIRT2	Oxidative stress and proliferation-apoptosis	Inhibit proliferation and induce apoptosis	subcutaneous model	[168]
C646	histone H3	Cell cycle	Inhibit tumour growth; Enhance antitumour efficacy	subcutaneous model	[169]
pyrrole-pyridine-imidazole derivative 8a	SIRT6	Apoptosis; DNA damage repair	Inhibit proliferation; Promote apoptosis; Reverse resistance to gemcitabine.	subcutaneous model	[170]
Givinostat; Dacinostat	HDAC	Anti-tumour immunity	Enhance the CTL-mediated killing	cell model	[171]
ISOX	HDAC6	Stem cell properties	Inhibit tumour growth and metastasis; Enhance the antitumor effect of 5-FU	tumor organoid model; orthotopic model	[172]
TAK-981	SUMO ligase E1	Cell cycle; Interferon signalling pathway	Impedes tumour progression; Enhance antitumour immune responses	subcutaneous model	[181]
ML-792; ML-93	SUMO ligase	Cell cycle; Apoptosis	Inhibit tumour growth and induce apoptosis	tumor organoid model; subcutaneous model	[184]

## 5. Challenges and Future Perspectives

Despite the compelling evidence supporting the critical roles of PTMs in PDAC pathogenesis and the encouraging development of targeted agents, translating these findings into effective clinical strategies faces considerable hurdles. To proceed, it is imperative to possess a comprehensive understanding of the challenges that lie ahead and to collectively endeavour to devise innovative solutions.

There are significant challenges in translating PTMs into clinical advances for PDAC. The dynamic, transient, and low-stoichiometry nature of many critical PTMs renders them notoriously difficult to detect and study within the complex in vivo environment. Conventional mass spectrometry frequently encounters difficulties in capturing the complete spectrum of modifications or deciphering the interplay between them within a single analysis.

Notably, the unique characteristics of pancreatic tissue samples and practical challenges in detection procedures further amplify the aforementioned difficulties, representing a critical bottleneck urgently requiring resolution for clinical translation. Pancreatic cancer tissues are often accompanied by severe fibrosis, necrosis, and cellular heterogeneity [203], hindering standardised sample processing. Meanwhile, the impact of preanalytical variables—including tissue ischemia time, discrepancies in fixation protocols, and variations in lysate preparation methods—cannot be ignored, as these factors may induce artificial PTM alterations, significantly reducing the reproducibility of detection results across different laboratories and clinical centres. To address these issues, a series of emerging technologies has demonstrated clear potential; for instance, enrichment chemistries can selectively isolate modified peptides from complex pancreatic tissue lysates, substantially enhancing the detection sensitivity of low-stoichiometry PTMs. Advanced mass spectrometry platforms integrated with data-independent acquisition (DIA) technology further optimise the reproducibility of quantitative analysis by ensuring consistent detection of modified peptides across diverse samples.

Moreover, the mere identification of a modification site is inadequate in itself; the precise definition of the functional consequences of a specific modification remains a formidable challenge, as exemplified by the distinct outcomes of STAT3 phosphorylation at Tyr705 versus Ser727 [43]. This underscores the critical need for tools such as site-specific antibodies and chemogenetic methods for functional dissection.

However, subtype-specific PTM functional differences offer a clear entry point for translating basic research into clinical practice. The translational potential of PTM-directed therapies in PDAC is constrained by the heterogeneity of tumour subtypes. Distinct PDAC subtypes (e.g., classical and basal-like) have unique PTM signatures: basal-like tumours show elevated STAT3 phosphorylation [204], while classical subtypes exhibit aberrant histone H3K27 acetylation [205]. These profiles enable patient stratification and precise matching of therapies to responsive populations. Reliable detection of site-specific PTMs in routine biopsies is critical, and several technologies show promise. Site-specific antibodies work well for known PTMs in formalin-fixed paraffin-embedded samples; activity-based probes target enzyme function. Imaging mass spectrometry (IMS) analyses PTM spatial distribution, and proximity labelling captures context-dependent PTM interactomes. These detection methods optimise PTM-directed therapy trials. Together, these strategies drive basic PTM-related mechanisms toward personalised PDAC care.

The inherent heterogeneity of the TME introduces an additional layer of complexity, as the PTM landscapes are likely to differ significantly between cancer cells, CAFs, and immune cells. The spatial resolution of current technologies is inadequate for the purpose of mapping PTMs across these cellular neighbourhoods at high throughput. Single-cell phosphoproteomics exhibits transformative potential in this field [206]. By integrating microfluidic cell sorting with ultra-sensitive mass spectrometry or proximity extension assays, this technology enables quantitative analysis of PTMs in individual cells within pancreatic tissues, directly resolving the spatial and cellular heterogeneity of PTM networks. The integration of spatial omics with proteomics is a pivotal future direction in addressing this knowledge gap and providing a more comprehensive understanding of the TME.

Therapeutic development is hindered by two main issues. Firstly, there is the question of drug specificity, and secondly, there is the issue of innate resistance. A significant proportion of PTM enzymes are members of large families that exhibit highly conserved catalytic domains. This characteristic renders the design of highly selective inhibitors challenging and frequently results in off-target toxicities. Strategies that focus on protein–protein interaction interfaces, as opposed to catalytic pockets, offer a promising path to enhanced specificity. Furthermore, the function of these enzymes is frequently embedded within large macromolecular complexes, suggesting that merely inhibiting catalysis may be inadequate—in this context, targeting the complex itself represents a new frontier. The most significant challenge is that of therapeutic resistance; for instance, the KRAS G12D inhibitor MRTX1133 has been found to trigger feedback activation across multiple pathways [73,74,75]. The plasticity of PTM networks necessitates a shift from monotherapy to rational combination strategies.

Addressing these challenges requires a concerted multidisciplinary effort focused on three interconnected priorities: driving technological innovation in spatial and functional PTM analysis, advancing therapeutics through novel specific inhibitors and rational combination therapies informed by network biology, and accelerating translation by defining PTM-driven molecular subtypes while developing non-invasive biomarkers. By systematically addressing these fronts, targeting PTMs holds the genuine potential to transform the therapeutic landscape and improve the dismal prognosis of PDAC.

## Abbreviations

The following abbreviations are used in this manuscript:

PTMs	Post-translational modifications
PDAC	Pancreatic ductal adenocarcinoma
EMT	Epithelial–mesenchymal transition
PNI	Perineural invasion
DSB	DNA double-strand break
TAMs	Tumour-associated macrophages
CAFs	Cancer-associated fibroblasts
TME	Tumour microenvironment
MDSCs	Myeloid-derived suppressor cells
PDX	Patient-derived xenografts
OXPHOS	Oxidative phosphorylation
UPS	Ubiquitin-proteasome system
USP	Ubiquitin-specific protease
UCH	Ubiquitin C-terminal hydrolase
OTU	Ovarian tumour protease
DUBs	Deubiquitinating enzymes
HAT	Histone acetyltransferase
SAE	SUMO ligase
Kla	Lactylation
pCAFs	Perineuronal infiltration-associated cancer-associated fibroblasts
OGTs	O-GlcNAcyltransferases
OGAs	O-GlcNAc hydrolases
IMS	Imaging mass spectrometry
DIA	Data-independent acquisition
ICBs	Immune checkpoint blockers
PDO	Patient-derived organoids
